# Inducing Ovulation with hCG Improves Fertility Outcomes of Co-Dominant Follicle Drainage to Avoid Twin Pregnancy in Dairy Cows

**DOI:** 10.3390/ani11010169

**Published:** 2021-01-12

**Authors:** Irina Garcia-Ispierto, Mònica Pando, Mònica Llobera-Balcells

**Affiliations:** 1 Agrotecnio Centre, University of Lleida, 25198 Lleida, Spain; 2 Department of Animal Science, University of Lleida, 25198 Lleida, Spain; monicalloberabalcells@gmail.com; 3 Granja Allué, Sucs, 25113 Lleida, Spain; monip_14@hotmail.com

**Keywords:** bovine, twins, therapeutics, heat stress, hormone treatment, synchronization

## Abstract

**Simple Summary:**

In dairy herds, twin pregnancies considerably compromise cow welfare, reducing their lifespan and leading to the increased use of antibiotics postpartum. In cows with follicles of pre-ovulatory size at insemination, twins can be avoided by puncturing and draining one follicle. In this report, we propose the use of a recently described manual method of follicular drainage without the need for ultrasound guidance and its association with treatment of the animal with human chorionic gonadotrophin (hCG), thus also increasing fertility compared to the use of Gonadotrophin releasing hormone (GnRH). The manual follicular drainage technique is simple, takes less than 1 min, and can be performed by a clinical veterinarian. In this study, even during heat stress periods follicle drainage plus hCG treatment gave rise to nearly 50% of pregnancies relative to conception rates, compared to 29% observed in non-drained, non-treated control animals or 20% in animals drained and treated with GnRH. This approach is recommended for weekly reproductive program visits.

**Abstract:**

Twin pregnancies are undesirable in dairy cattle as they dramatically compromise cow lifespan and, consequently, herd economy. Clinical problems in cows arise from the time of pregnancy diagnosis to pregnancy loss, abortion, or parturition. The drainage of co-dominant follicles in cows with two or more follicles at insemination prevents twin pregnancy. The objective of this study was to compare the effectiveness of draining the smaller of two follicles through a simplified maneuver not requiring ultrasonography in cows in their third or more lactation, and then inducing ovulation immediately before artificial insemination (AI) with GnRH or human chorionic gonadotrophin (hCG). Animals were monitored by ultrasound at AI and randomly assigned to the groups: follicular drainage and treatment with GnRH (Deph; *n* = 60), follicular drainage and treatment with hCG (hCG; *n* = 60), and non-drainage (ND; *n* = 60) as control cows. On the basis of odds ratios, cows treated with hCG were 2.1 times more likely to become pregnant than control animals. Our results reveal the efficacy of hCG treatment at AI in cows with two follicles of pre-ovulatory size subjected to a simple follicular drainage procedure.

## 1. Introduction

When a dairy cow becomes pregnant, maintaining this pregnancy is more important than fertility for herd economy. Thus, for dairy producers, pregnancy loss (PL) is a key factor to be avoided and losses during the first trimester of gestation in high-producing dairy herds may exceed 20% [1,2]. Once an infectious cause has been ruled out, PL can be attributed to several non-infectious factors [3]. One of the most significant of these factors is twin pregnancy [2]. Over the years, twin pregnancy rates have increased [4], probably because of an increasing double ovulation rate associated with increased milk production [5].

Ultrasound-guided puncture and drainage without suction of co-dominant follicles at artificial insemination (AI) has recently proved to be an efficient procedure to eliminate the risk of twin pregnancy without reducing fertility [6,7]. This technique avoids the risk of pregnancy loss related to induced twin reduction and increases the incidence of additional corpora lutea. Recently, a transvaginal follicular drainage technique without ultrasound has been validated for drainage of co-dominant follicles with no suction at AI for twin pregnancy prevention in cows with two follicles of pre-ovulatory size [8]. This simplified method is easy for experienced reproductive veterinarians and can be performed in less than one minute per cow. Although fertility is similar to that of monovular non-treated animals and pregnancy losses are clearly reduced, there remains a high risk of ovulation failure in the non-drained follicle. By administering a more potent inductor of ovulation at AI this risk of ovulation failure could be reduced and this approach could even increase the fertility of drained biovular cows.

Human chorionic gonadotrophin (hCG) binds directly to LH receptors [9] inducing ovulation and exerting a luteotropic effect [10]. Additionally, unlike Gonadotrophin releasing hormone (GnRH)-induced ovulation which causes negative feedback of systemic progesterone (P4) concentrations [11,12], ovulation induced by hCG is independent of the pituitary gland. Hence, the administration of hCG at AI should help reduce ovulation failure in cows undergoing follicle drainage.

The present study compares the effectiveness of GnRH and hCG given at AI in inducing ovulation of the non-drained follicle in cows with two follicles of pre-ovulatory size undergoing drainage of the smaller follicle through a new simple non ultrasound-guided procedure.

## 2. Materials and Methods

**Figure 1:**
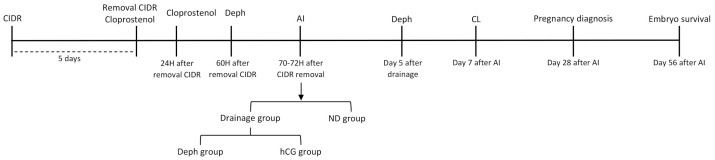
Fixed-time artificial insemination protocol and experimental design used in 180 cows (AI: artificial insemination; CL: corpus luteum observations; Deph: Dephereline; ND: non-drainage group).

This study was performed over the period January to September 2020 in a commercial dairy herd of Holstein-Friesian cows located in northeastern Spain (Lleida). Cows were enrolled if they had a body condition score of 2.5–3.5 on a scale 1 to 5 [13], produced more than 30 kg milk per day, and were free of detectable reproductive disorders and/or clinical disease during the study period (from 5 days before to 60 days after insemination). Cows were selected for a fixed-time insemination AI (FTAI) protocol [14] (Figure 1) and first treated with a controlled internal drug-releasing device (CIDR) (CIDR, containing 1.38 g of progesterone; Zoetis Spain SL, Alcobendas, Madrid, Spain). The CIDR was left in place for 5 days, and these animals were also given cloprostenol (500 μg intramuscular (i.m.); PGF Veyx Forte, Ecuphar, Barcelona, Spain) on CIDR removal. Then, 24 and 60 h later, the cows received a second cloprostenol and GnRH dose (using the GnRH analogue dephereline: 100 μg gonadorelin acetate [6-D-Phe] i.m.; Gonavet Veyx, Ecuphar, Barcelona, Spain), respectively, and were subjected to FTAI 70–72 h after CIDR removal. A cow was confirmed to be in estrus and therefore ready for insemination both by ultrasonography (using a portable B-mode ultrasound scanner equipped with a linear 5–10 MHz transvaginal transducer) and manual rectal palpation [15]. Only cows either with two bilateral or unilateral co-dominant follicles over 12 mm in diameter in the absence of a corpus luteum were included in the study. Further prerequisites were a uterus that was highly tonic and contractile to the touch, and a vaginal discharge of copious clear fluid. As double ovulation has been related to the least possible size difference between the larger and smaller follicle irrespective of the individual diameter of each follicle [16], only cows in their third lactation or more showing a follicular size difference of less than 2 mm between the 2 co-dominant follicles were included in the study.

### 2.1. Follicular Drainage and Experimental Design

Cows with unilateral or bilateral follicles were randomly assigned to the groups: follicular drainage plus Dephereline treatment (Deph; *n* = 65), follicular drainage plus hCG treatment (Veterin Corion 750 UI/mL, Divasa-Farmavic, Gurb-Vic, Barcelona, Spain) (hCG; *n* = 65), and controls neither drained nor treated (ND; *n* = 65). In cows in the follicle drainage groups, the smaller follicle was punctured and drained with the help of a steel transvaginal cannula designed for follicular cyst puncture [8]. Immediately before follicular puncture, the cannula, vulva, and perineal region of the cow were washed with disinfectant solution. Ovaries bearing subordinate follicles were positioned adjacent to wall by rectal manipulation. The end of the cannula (outer cannula sleeve: 1.2 cm outer diameter/50 cm length) was introduced into the dorsal vaginal fornix, to the left or right of the cervix depending on the follicle to be punctured. The follicle was then positioned transrectally against the flat end of the cannula sleeve so that the follicle was separated only by the vaginal wall. The inner cannula (0.8 cm outer diameter/49.5 cm length) fitted with a sterile 18G 25-mm long needle was inserted in the ovary, and the vaginal wall was then pierced in a cranial direction through the fornix with the needle introduced into the follicular antrum as previously described [6]. No suction was applied. Cows underwent AI immediately after follicle puncture and drainage followed by a Dephereline or hCG dose. Further, the function of drainage-induced corpus luteum (CL) was improved with GnRH treatment on day 5 post-drainage [7].

Ovulation was recorded as the presence of a CL assessed 7 days post-AI. On day 7 post-AI, 10 cows were withdrawn from the study as they failed to ovulate (2 in non-drainage (ND), 4 in hCG, and 5 in Deph). In drained animals, ovulation failure was defined as the absence of a CL in the non-drained ovary 7 days after drainage. All data were calculated for ovulating animals. This resulted in a final study population of 63, 61, and 60 cows in the ND, hCG, and Deph groups, respectively. Pregnancy diagnosis was performed by ultrasonography 28 days post-AI and embryo survival assessed at 56 days post-AI. The pregnancy rate was defined as the percentage of cows that were confirmed pregnant 28 days after FTAI out of the total number of cows in the corresponding group. The maximum temperature–humidity index (THI) on the day of FTAI was used to evaluate the effects of heat stress (HS) (THI values higher than 72) [17] on subsequent reproductive performance.

The maximum THI index was fitted to the equation [18]:

Maximum THI = (0.8 × maximum T + minimum RH (%)/100 × (maximum T − 14.4) + 46.4)

It should be noted that in our geographical region, a clear negative effect of heat stress (HS) on the reproductive performance of lactating dairy cows has been described [14,19].

All procedures were approved by the Ethics Committee on Animal Experimentation of the University of Lleida (license number CEEA.06-01/12).

### 2.2. Data Collection and Statistical Analysis

The following data were recorded for each animal: parturition and treatment dates; lactation number (>3 lactations); treatment (Deph, hCG, ND); follicular size (mm) at FTAI; maximum THI at treatment (≤72 versus >72); milk production at AI (low producers <45 kg versus high producers ≥45 kg); days in milk at AI (≤90 days versus >90 days); repeat breeder syndrome (<4 AI vs. ≥4 AI at treatment); number of CL and size (mm) 7 days after FTAI; ovulation failure (absence of a CL 7 days post-AI); sire; AI technician; conception rate 28 days post-AI; presence of twins; and pregnancy loss at 56 days post-AI.

The overall reproductive performance of the cows by treatment group was assessed through binary logistic regression (PASW Statistics for Windows Version 18.0 (SPSS Inc., Chicago, IL, USA). Four binary logistic regression models were built in which ovulation failure, conception rate, presence of twins after FTAI, and pregnancy loss were the dependent variables. The factors entered in the model as independent dichotomous variables (where 1 denotes presence and 0 denotes absence) were HS at treatment (THI > 72), milk production (<45 kg vs. ≥45 kg), repeat breeding syndrome (<4 AI vs. ≥4 AI), and days in milk (<90 days vs. ≥90 days after parturition). Treatment (ND, hCG, and Deph), AI technician, and sire (class variables) were considered factors in the analyses. Possible interactions between treatment and dichotomous variables were also examined. For the dependent variable conception rate, only ovulating animals were considered. For the dependent variables twin pregnancy and pregnancy loss, only pregnant cows were considered.

Regression analyses were conducted according to the method of Hosmer and Lemeshow [20] using the logistic procedure of PASW Statistics for Windows Version 18.0 (SPSS Inc., Chicago, IL, USA). Essentially, this method consists of 5 steps as follows: preliminary screening of all variables for univariate associations, construction of a full model using all the significant variables arising from the univariate analysis, stepwise removal of non-significant variables from the full model and comparison of the reduced model with the previous model for model fit and confounding, evaluation of plausible interactions among variables, and assessment of model fit using Hosmer–Lemeshow statistics. Variables with univariate associations showing *p*-values < 0.25 were included in the initial model. Model reduction continued until only significant terms according to the Wald statistic remained in the model at *p* < 0.05.

## 3. Results

Mean milk production at treatment, days in milk at AI, number of lactations, and number of inseminations were 48.7 ± 8.7 kg, 140 ± 77 days, 3.6 ± 1.6 lactations, and 3.1 ± 2.3 inseminations, respectively (mean ± SD). The independent variables recorded for each of the three treatment groups and the effects of the different treatments on conception rate, twin pregnancies, and pregnancy loss are shown in Table 1.

Of the 183 cows included in the study, 59 (32.2%) became pregnant: 18 (28.5%), 29 (47.5%) and 12 (20.0%) in the ND, hCG, and Deph groups, respectively. Table 2 shows odds ratios for the factors affecting conception rate after treatment included in the final logistic regression model. Accordingly, cows treated with hCG were 2.1 times more likely to become pregnant than non-drained controls.

No twin pregnancies were recorded in any animal in the two drained groups. Of the 18 pregnancies in the ND group, 5 were twin pregnancies. No factor was related to ovulation failure or pregnancy loss.

## 4. Discussion

This study was designed to examine the rates of ovulation failure, conception rate, twin pregnancies, and subsequent pregnancy loss in response to treatment with GnRH or hCG at AI in cows with two follicles of pre-ovulatory size at AI subjected to a simple follicular drainage method. It should be highlighted that this procedure was performed during a weekly reproductive control program and only in multiparous cows. These animals are the higher producers and the most susceptible to twin pregnancy [21,22].

No twin pregnancies were recorded in any of the study participants. This means that by examining all animals at AI and then subjecting them to follicular drainage of the subordinate follicle twin pregnancy was avoided, which is especially common in multiparous animals [8]. Clinical veterinarians should accordingly modify their AI procedure to improve cow welfare and avoid reduction of twins, especially in well managed high producing dairy herds. The protocol proposed in this study can be performed in commercial farms without interfering with reproduction program visits and is also a cost-saving measure. Reducing the incidence of double deliveries will both benefit herd economy and animal welfare.

No differences in ovulation failure were detected among the treatment groups, although cows receiving hCG were 2.1 times more likely to conceive than others. Although hCG and GnRH have similar effects on the ovary [23,24], the good conception response to hCG could mean that this hormone not only acts directly on the ovarian follicle, as opposed to GnRH, which acts via the hypophyseal-ovarian axis [10], but also on the CL, reducing early embryo losses that would pass unnoticed in a herd. Further, GnRH treatment induces an LH surge lasting about 5 h [25], which is approximately half the duration of a naturally occurring LH surge [26]. This may suggest that hCG, besides appropriately inducing ovulation in drained animals, also improves CL health, increasing plasma progesterone concentrations [27,28]. The hypothesis that hCG is able to increase the conception rate by avoiding ovulation failure seems not feasible. More work is needed to determine the effect of hCG after simplified follicular drainage.

In drained animals, the number of corpora lutea exceeded the number of embryos. It is well known that this is a strong factor promoting pregnancy maintenance [15], as progesterone is essential for implantation and placentation [29]. Considering that no twin pregnancies were recorded in the two study groups, the drainage of subordinate follicles added to an inductor of ovulation seems not only able to avoid twin pregnancies, but also losses of single pregnancies.

Another factor typically affecting the conception rate in our geographical area, heat stress, was not found relevant. It is likely that the beneficial effects of treatment outstripped the negative effects of heat stress or repeat breeding syndrome. Notably, the conception rate of 48% included the warm season with the THI reaching 85 units.

## 5. Conclusions

The results of this study indicate that the fertility of cows with two follicles subjected to non-ultrasound guided follicular drainage plus hCG treatment at AI can be substantially improved, even during heat stress periods.

## Figures and Tables

**Table 1:** Independent variables recorded at the time of treatment and effects of treatment on conception rate, twin pregnancies, and pregnancy losses.

Variables	ND	hCG	Deph
Independent			
Milk production (≥45 kg)	39/63 (61.9)	35/61 (57.3)	36/60 (60.0)
Heat stress (>72 units) Repeat breeding syndrome ^a^ 2 CL 7 days post-AI	23/63 (36.5) 10/63 (15.8) 31/63 (49.2) ^a^	23/61 (37.7) 11/61 (18.0) 61/61 (100) ^b^	24/60 (40.0) 12/60 (20.0) 59/60 (98.3) ^b^
Dependent			
Conception rate ** Twin pregnancies *** Pregnancy losses ***	18/63 (28.6) ^a^ 5/18 (27.8) 1/5 (20.0)	29/61 (47.5) ^b^ 0 0	12/60 (20.0) ^a^ 0 0

^a,b^ Values with different superscripts differ within rows according to Tukey–Kramer tests (*p* < 0.05). ** Relative to ovulating cows. *** Relative to pregnant animals. ^a^ Repeat breeders > 3 AI.

**Table 2:** Odds ratios of the variables included in the final logistic regression model for factors affecting conception rate after treatment.

Factor	Class	*n*	% Conception Rate	Odds Ratio	95% Confidence Interval	*p*
Treatment *	ND hCG GnRH	18/63 29/61 12/60	28.6 47.5 20.0	Reference 2.1 0.5	1.1–4.1 0.2–1.1	0.008 0.1

* ND: no follicular drainage; human chorionic gonadotrophin (hCG): follicular drainage plus 3000 IU hCG; Gonadotrophin releasing hormone (GnRH): follicular drainage plus 100 µg GnRH. Hosmer and Lemeshow goodness-of-fit-test = 25.6; 3 degrees of freedom (df), *p* = 0.90, *R*^2^ Nagelkerke = 0.12.

## Data Availability

The data presented in this study are available on request from the corresponding author. The data are not publicly available to preserve privacy of the data of the commercial farm.

## References

[1] Grimard B., Freret S., Chevallier A., Pinto A., Ponsart C., Humblot P. (2006). Genetic and environmental factors influencing first service conception rate and late embryonic/foetal mortality in low fertility dairy herds. Anim. Reprod. Sci..

[2] López-Gatius F., Szenci O., Bech-Sàbat G., García-Ispierto I., Serrano B., Santolaria P., Yániz J. (2009). Factors of non-infectious nature affecting late embryonic and early foetal loss in high producing dairy herds in north-eastern Spain. Magy. Allatorv. Lapja.

[3] Vanroose G., de Kruif A., Van Soom A. (2000). Embryonic mortality and embryo-pathogen interactions. Anim. Reprod. Sci..

[4] López-Gatius F., Andreu-Vázquez C., Mur-Novales R., Cabrera V.E., Hunter R.H.F. (2017). The dilemma of twin pregnancies in dairy cattle. A review of practical prospects. Livest. Sci..

[5] Fricke P.M., Wiltbank M.C. (1999). Effect of milk production on the incidence of double ovulation in dairy cows. Theriogenology.

[6] López-Gatius F., Hunter R.H.F. (2018). Puncture and drainage of the subordinate follicles at timed artificial insemination prevents the risk of twin pregnancy in dairy cows. Reprod. Domest. Anim..

[7] López-Gatius F., Garcia-Ispierto I., Serrano-Pérez B., Balogh O.G., Gábor G., Hunter R.H.F. (2019). Luteal activity following follicular drainage of subordinate follicles for twin pregnancy prevention in bi-ovular dairy cows. Res. Vet. Sci..

[8] Garcia-Ispierto I., López-Gatius F. (2020). Improved embryo survival following follicular drainage of subordinate follicles for twin pregnancy prevention in biovular dairy cows. J. Reprod. Dev..

[9] Binversie J.A., Pfeiffer K.E., Larson J.E. (2012). Modifying the double-Ovsynch protocol to include human chorionic gonadotropin to synchronize ovulation in dairy cattle. Theriogenology.

[10] De Rensis F., López-Gatius F., García-Ispierto I., Techakumpu M. (2010). Clinical use of human chorionic gonadotropin in dairy cows: An update. Theriogenology.

[11] Atkins J.A., Busch D.C., Bader J.F., Keisler D.H., Patterson D.J., Lucy M.C., Smith M.F. (2008). Gonadotropin-releasing hormone-induced ovulation and luteinizing hormone release in beef heifers: Effect of day of the cycle. J. Anim. Sci..

[12] Dias F.C., Colazo M.G., Kastelic J.P., Mapletoft R.J., Adams G.P., Singh J. (2010). Progesterone concentration, estradiol pretreatment, and dose of gonadotropin-releasing hormone affect gonadotropin-releasing hormone-mediated luteinizing hormone release in beef heifers. Domest. Anim. Endocrinol..

[13] Edmondson A.J., Lean I.J., Weaver C.O., Farver T., Webster G. (1989). A body condition scoring chart for Holstein dairy cows. J. Dairy Sci..

[14] Garcia-Ispierto I., López-Gatius F. (2014). Effects of different five-day progesterone-based fixed-time AI protocols on follicular/luteal dynamics and fertility in dairy cows. J. Reprod. Dev..

[15] López-Gatius F. (2012). Factors of a noninfectious nature affecting fertility after artificial insemination in lactating dairy cows. A review. Theriogenology.

[16] López-Gatius F., Garcia-Ispierto I., Serrano-Pérez B., Hunter R.H.F. (2018). The presence of two ovulatory follicles at timed artificial insemination influences the ovulatory response to GnRH in high-producing dairy cows. Theriogenology.

[17] De Rensis F., Garcia-Ispierto I., López-Gatius F. (2015). Seasonal heat stress: Clinical implications and hormone treatments for the fertility of dairy cows. Theriogenology.

[18] Thom E.C. (1959). The discomfort index. Weatherwise.

[19] López-Gatius F. (2003). Is fertility declining in dairy cattle? A retrospective study in northeastern Spain. Theriogenology.

[20] Hosmer D.W., Lemeshow S. (1989). Applied Logistic Regression.

[21] Jones S.V.H., Rouse J.R. (1920). The relation of age of dam to observed fecundity in domesticated animals. I. Multiple births in cattle and sheep. J. Dairy Sci..

[22] Fitzgerald A.M., Berry D.P., Carthy T., Cromie A.R., Ryan D.P. (2014). Risk factors associated with multiple ovulation and twin birth rate in Irish dairy and beef cattle. J. Anim. Sci..

[23] Sianangama P.C., Rajamahendran R. (1992). Effect of human chorionic gonadotropin administered at specific times following breeding on milk progesterone and pregnancy in cows. Theriogenology.

[24] Fricke P.M., Reynolds L.P., Dale A.R. (1993). Effects of human chorionic gonadotropin administered early in estrus cycle on ovulation and subsequent luteal function in cows. J. Anim. Sci..

[25] Chenault J.R., Kratzer D.D., Rzepkowski R.A., Goodwin M.C. (1990). LH and FSH response of Holstein heifers to fertilerin acetate, gonadorelin and buserelin. Theriogenology.

[26] Rahe C.H., Owens R.E., Fleeger J.L., Newton H.J., Harms P.G. (1980). Pattern of plasma luteinizing hormone in the cyclic cow: Dependent upon the period of the cycle. Endocrinology.

[27] Maillo V., Duffy P., O’Hara L., de Frutos C., Kelly A.K., Lonergan P., Rizos D. (2014). Effect of hCG administration during corpus luteum establishment on subsequent corpus luteum development and circulating progesterone concentrations in beef heifers. Reprod. Fertil. Dev..

[28] Price C.A., Webb R. (1989). Ovarian response to hCG treatment during the oestrous cycle in heifers. J. Reprod. Fertil..

[29] Spencer T.E., Johnson G.A., Bazer F.W., Burghart R.C., Palmarini M. (2007). Pregnancy recognition and conceptus implantation in domestic ruminants: Roles of progesterone, interferons and endogenous retroviruses. Reprod. Fertil. Dev..

